# The Yellow Fever in San Antonio

**Published:** 1903-12

**Authors:** A. S. McDaniel

**Affiliations:** San Antonio, Texas


### Correspondence.

### The Yellow Fever in San Antonio.

LETTERS FROM DRS. M'DANIEL, LLOYD, BLEIM, SPRING, SHROPSHIRE AND PASCHAL.

SAN ANTONIO, Texas, November 27, 1903.

*Editor Texas Medical Journal:*

Some doubt has been expressed in the lay press of this city as to the correctness of the diagnosis made by Drs. Murray, Tabor and myself in the case of Mrs. Kurka, who died at 643 Rembrano street, this city, on October 11th. I therefore beg to submit the following brief statement concerning this case and the circumstances surrounding and immediately following her death:

Mrs. Kurka, aged 26 years, healthy and strong, had not had any malarial troubles, was taken sick on the 6th day of October, with a temperature of 104°, pulse 60, eyes injected, constant nausea and vomiting. This condition continued, with suppression of urine about the fourth day, vomit black and death on the 11th, five days after the attack.

Her mother, who came from Hondo, a non-malarial country, and nursed her daughter from Thursday until Sunday, went back with the body to their home, and was taken sick four days afterward, and ran the course of all the symptoms of yellow fever, dying with black vomit on the sixth or seventh day. This is the case that Dr. Tabor was called from Laredo to see. The husband of the mother was taken sick a week afterward, and died with all the symptoms of yellow fever, this making three of one family from the same house who died. Mrs. Urbeneck, living directly across the street from Mrs. Kurka, and who was nursing her before her mother came, was taken sick five days after Mrs. Kurka's death, and died with all the symptoms of yellow fever, so pronounced by her attending physician, Dr. Braunagel. A boy by the name of Adams, 16 years old, living one block below Mrs. Kurka, died about a week after her death, and there was black vomit all over the bed clothes, as stated by neighbors. A girl that lived at West End, and came by Mrs. Kurka's every morning to her work in town, was taken sick and died about ten days after Mrs. Kurka's death, at the City Hospital. A post mortem was made of this case, that showed every change that occurs from yellow fever, making it out absolutely, without any doubt, a genuine case.

Then, to repeat, Mrs. Kurka died at 643 Rembrano street; her mother came to nurse her and died; her father died; Mrs. Urbeneck, who waited on her first, died; the girl, who came by her house every morning, died, and a 16-year-old boy, who lived one block from her, died, all from one source of infection, and all with genuine yellow fever.

A. S. McDaniel, M. D.,

[It seems that Mrs. Kurka's mother nursed her daughter four days, and developed fever *four days later.* When did the mosquito get in his work? It requires not less than *seventeen days* by the mosquito route.—Editor].

